# Dynamic field mapping and distortion correction using single-shot blip-rewound EPI (rEPI) and joint multi-echo reconstruction

**DOI:** 10.1002/mrm.30038

**Published:** 2024-02-02

**Authors:** Wenchuan Wu

**Affiliations:** 1 Wellcome Centre for Integrative Neuroimaging, FMRIB, Nuffield Department of Clinical Neurosciences, University of Oxford, Oxford, United Kingdom

**Keywords:** EPI, distortion correction, dynamic field mapping, fMRI, motion

## Abstract

**Purpose:**

To develop a method for dynamic ΔB_0_ mapping and distortion correction.

**Methods:**

A blip-rewound EPI trajectory was developed to acquire multiple 2D EPI images in a single readout with an interleaved order, which allows a short echo time difference. A joint multi-echo reconstruction was utilized to exploit the shared information between EPI images. The reconstructed images from each readout are combined to produce a final magnitude image. A ΔB_0_ map is calculated from the phase of these images for distortion correction. The efficacy of the proposed method is assessed with phantom and in vivo experiments. The performance of the proposed method in the presence of subject motion is also investigated.

**Results:**

Compared to conventional multi-echo EPI, the proposed method allows dynamic ΔB_0_ mapping at matched resolution with a much shorter TR. Phantom and in vivo results show that the proposed method can provide a comparable magnitude image as conventional single-shot EPI. The ΔB_0_ maps calculated from the proposed method are consistent with conventional multi-echo EPI in the phantom experiment. For in vivo experiments, the proposed method provides a more accurate estimation of ΔB_0_ than conventional multi-echo EPI, which is prone to phase wrapping problems due to the long echo time difference. In-vivo scan with subject motion shows the proposed dynamic field mapping method can improve the temporal stability of EPI time series compared to GRE-based static field mapping.

**Conclusion:**

The proposed method allows accurate dynamic ΔB_0_ mapping for robust distortion correction without compromising spatial or temporal resolution.

## Introduction

Single-shot Echo planar imaging (EPI) is the workhorse for functional MRI (fMRI) studies due to its fast acquisition speed that enables high sensitivity to blood oxygenation level dependent (BOLD) contrast^1^. However, because of the low bandwidth along the phase encoding direction, single-shot EPI is prone to B_0_ field inhomogeneity, which arises from local susceptibility variations at the tissue-air and tissue-bone interfaces, as well as hardware imperfections. B_0_ field inhomogeneity can cause geometric distortions in EPI images along the phase encoding direction, leading to incorrect localization of signals.

Conventional EPI distortion correction measures a map of local B_0_ field offsets ΔB_0_ before or after EPI acquisition^2^ and it assumes the B_0_ inhomogeneity does not change over the course of the EPI scan. However, this static field mapping method cannot capture dynamic B_0_ field changes due to subject motion, respiration, gradient heating, and eddy currents^3^. The uncompensated B_0_ field fluctuations can result in temporally varying image distortions, which may obscure the subtle activation signals we aim to detect with fMRI.

A number of dynamic field mapping methods have been proposed to address the limitation of static field mapping. One strand of methods acquires navigators to estimate a ΔB_0_ map for each EPI image volume. Different navigator acquisition techniques have been developed, such as the double-echo volumetric navigator^4^, Cloverleaf navigator^5^, and FID navigator^6,7^. Navigator acquisition requires a longer scan time and reduces the temporal resolution of the EPI time series. Some navigator-based methods (e.g., Cloverleaf navigator) also increase the minimal achievable echo time (TE) compared to the standard EPI sequence. The FID navigator is an efficient approach that imposes negligible impact on the temporal resolution of the EPI time series. However, it requires contrast-matched calibration data to fit the signal model, which needs extra scan time. ΔB_0_ can also be measured using an external NMR field probe^8^, which provides real-time field monitoring. However, the requirement of extra hardware may hinder its wide application.

Several navigator-free dynamic ΔB_0_ mapping methods have also been developed. One type of navigator-free method uses the conventional EPI readout and acquires one EPI image after each excitation. A dynamic ΔB_0_ map is calculated from the phase of each image volume by subtracting a phase offset term (due to non-B_0_ related contribution, e.g., B_1_ field inhomogeneity) from the image phase and then dividing the residual phase by the TE^9–13^. These methods typically require acquisition of a reference ΔB_0_ map using conventional multi-echo gradient echo sequence and use phase extrapolation to obtain reliable phase offset estimation when subject motion is large^12^.

Another type of navigator-free dynamic ΔB_0_ mapping method calculates a ΔB_0_ map using multiple EPI images acquired with different TEs^14–16^. The single-shot multi-echo EPI (me-EPI) based method acquires multiple EPI images following each excitation, which can be used to calculate a 2D ΔB_0_ map^15^. As each ΔB_0_ map is calculated from a single readout, this method is highly robust against subject motion and respiration. However, conventional me-EPI based dynamic ΔB_0_ mapping is limited in spatial and temporal resolution due to the acquisition of multiple EPI images. Parallel imaging techniques^17,18^ can mitigate these limitations by acquiring under-sampled k-space for each EPI image^15^, but are prone to SNR penalties and residual artifacts at high under-sampling factors, which are needed to achieve high spatial and temporal resolution.

This work presents a new method for dynamic ΔB_0_ mapping and EPI distortion correction by integrating a single-shot blip-rewound EPI (rEPI) trajectory, which acquires multiple 2D EPI images in a single readout with an interleaved order, and a joint multi-echo reconstruction, which exploits the shared information between EPI images from the same readout. By interleaving the acquisition of multiple EPI images (instead of using the sequential acquisition in conventional me-EPI), rEPI can acquire multiple EPI images with a short echo time difference (ΔTE), which allows robust dynamic ΔB_0_ mapping without compromising the spatial and temporal resolution. The multiple 2D EPI images acquired from a single readout are combined to produce a final magnitude image. Distortion correction is performed for each EPI volume based on estimated dynamic ΔB_0_ maps. The performance of the proposed method is assessed with respect to the conventional multi-echo EPI and GRE based field mapping using phantom and in vivo experiments.

## Methods

### Dynamic ΔB_0_ mapping with single-shot multi-echo EPI

**Figure 1:**
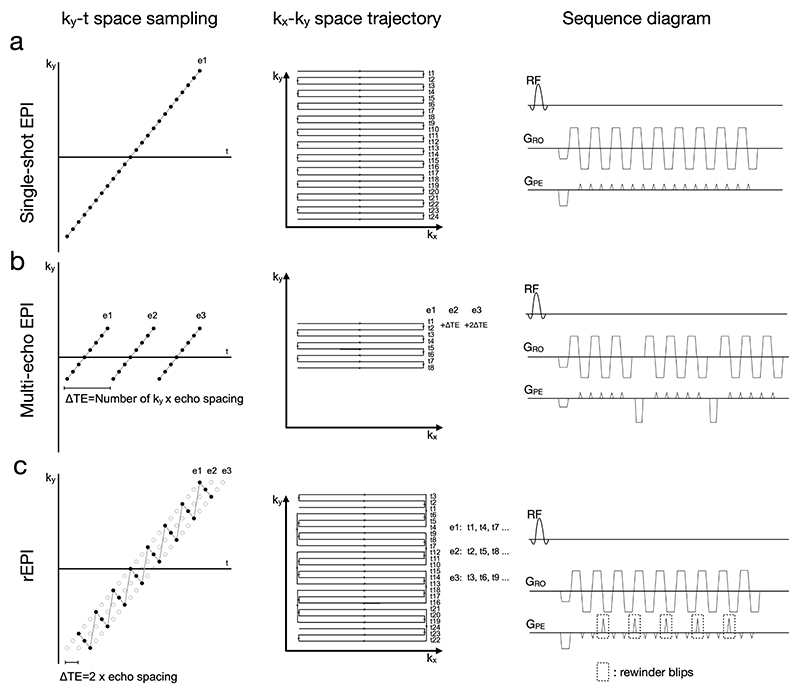
Illustration of different single-shot EPI sampling trajectories in k_y_-t space, k_x_-k_y_ space (i.e., k space), and the corresponding sequence diagrams. Each dot in the k_y_-t space represents a readout line. (a) conventional single-shot EPI. (b) Single-shot multi-echo EPI acquiring three echoes (echo 1: ‘e1’, echo 2: ‘e2’, echo 3: ‘e3’). (c) Single-shot blip-rewound EPI (rEPI) acquiring three echoes. To achieve the same TR as conventional single-shot EPI, single-shot multi-echo EPI needs to shorten the readout duration for each EPI image, leading to reduced spatial resolution. Different from single-shot EPI and single-shot multi-echo EPI, rEPI trajectory traverses k-space with a periodic rewinding pattern using k_y_ rewinder blips. t# denotes the acquisition order of EPI readout lines.

The local magnetic field inhomogeneity ΔB_0_(r) (in Hz) can be measured from the phase evolution ΔΦ(r) between two images acquired at different TEs ^2^:

Eq.1 $${\text{ΔB}}_{0}(\text{r})=\frac{\text{ΔΦ}(\text{r})}{2\text{πΔTE}}$$

where ΔTE is the TE difference between the two images. Conventional single-shot EPI acquires one EPI image with a single TE, and the corresponding sampling trajectory in the k_y_-t space is a single straight line (Fig. 1a). The single-shot multi-echo EPI (me-EPI) method ^15^ sequentially acquires multiple EPI images with different TEs following each excitation, corresponding to several straight lines in the k_y_-t space (Fig. 1b) separated by ΔTE, which is governed by the number of k_y_ encoding lines for each EPI image and the EPI echo spacing. With single-shot me-EPI, a 2D dynamic ΔB_0_ map can be estimated with Eq. 1 using data acquired from a single readout, providing high robustness against motion and respiration.

However, due to the sequential acquisition of multiple EPI images, ΔTE in me-EPI couples with the number of the k_y_ encoding lines. Therefore, the TR of me-EPI needs to be longer than conventional single-shot EPI with matched spatial resolution and field of view, which leads to reduced temporal resolution. Alternatively, to maintain the same temporal resolution as conventional single-shot EPI, the readout length of me-EPI must be shortened, which limits the spatial resolution (Fig. 1b).

### Dynamic ΔB_0_ mapping with single-shot blip-rewound EPI

#### Blip-rewound EPI trajectory

A single-shot blip-rewound EPI trajectory (rEPI) is developed to overcome the limitations of me-EPI. Like me-EPI, rEPI acquires multiple 2D EPI images with a single readout. Instead of using the sequential acquisition as in conventional me-EPI, rEPI acquires multiple EPI images with an interleaved order. Fig. 1c shows an rEPI trajectory that acquires three EPI images in a single readout, with the corresponding ΔTE being twice the EPI echo spacing. In k_y_-t space, the rEPI trajectory samples three straight lines that are temporally adjacent to each other using a zig-zag pattern. In k_x_-k_y_ space (i.e., k space), the rEPI trajectory traverses along the phase encoding direction with a periodic rewinding pattern, which is achieved by inserting periodic rewinder blips between standard EPI blips along the k_y_ phase encoding direction (Fig.1c, sequence diagram).

With the interleaved acquisition, rEPI de-couples the number of k_y_ encoding lines with ΔTE, enabling multiple EPI images to be acquired using the same readout duration as conventional single-shot EPI without compromising the spatial or temporal resolution (i.e., same k-space coverage in Fig. 1a and Fig. 1c). In addition, the short ΔTE in rEPI reduces the phase wrapping in ΔΦ(r), which can facilitate robust ΔB_0_ estimation.

#### Joint multi-echo reconstruction

Individual EPI images acquired with rEPI are under-sampled by a factor equal to the number of echoes multiplied by the parallel imaging under-sampling factor. Conventional parallel imaging reconstruction^17,18^ of each EPI image may be prone to g-factor noise amplification and residual aliasing artifacts. Due to the short ΔTE of rEPI acquisition, the EPI images acquired from an rEPI readout are expected to be highly similar, which can be leveraged to improve image reconstruction. Here, a joint multi-echo reconstruction based on low-rank Hankel structured matrix recovery is used^19–24^. To exploit the shared information between EPI images acquired with an rEPI readout, the Hankel-structured matrix is extended from a multi-coil construction as used in previous works to a multi-coil, multi-echo construction. The reconstruction problem is formulated as:

Eq.2 $$\begin{array}{c} x=\underset{x}{\arg\min}\|Ax-b{\|}_{l_{2}}^{2}\\ \;s.t.\;rank(H(x))=r \end{array}$$

**Figure 2:**
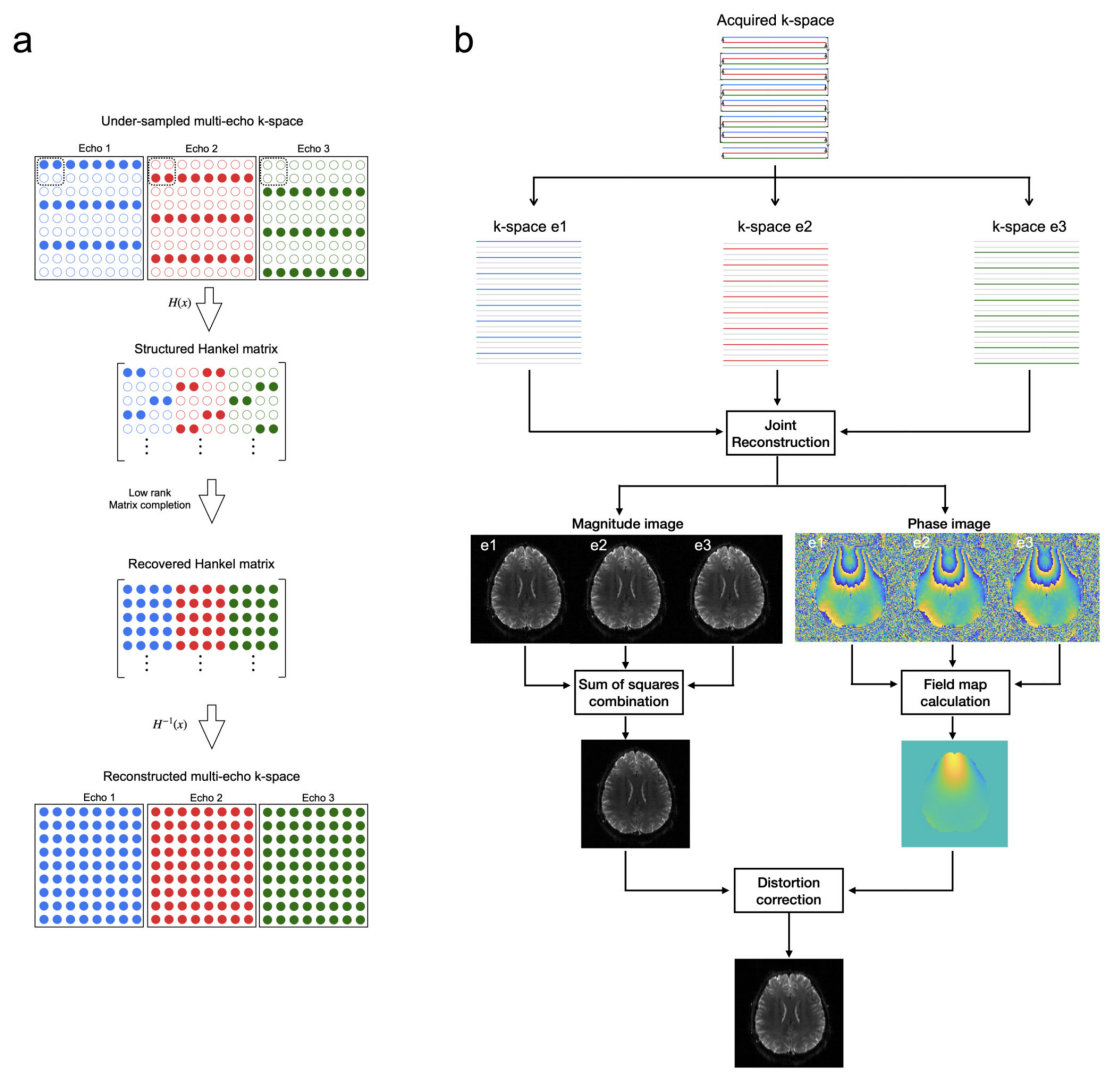
a). Diagram of low-rank Hankel structured matrix recovery. Solid and hollow circles denote the acquired and unacquired k-space data. b). Reconstruction and distortion correction pipeline of the proposed method. The acquired k-space is divided into three separate k-space, each corresponding to one echo image with difference TE (echo 1: ‘e1’, echo 2: ‘e2’, echo 3: ‘e3’). The three k-space were jointly reconstructed using a structured low-rank constrained reconstruction. Magnitude images from all echoes were combined to generate a final magnitude image. Phase images from all echoes were used to calculate a ΔB_0_ map. Distortion correction is performed on the combined magnitude image with the field offset information from the ΔB_0_ map.

Where *x* (*N*_*e*_*N*_1_*N*_2_ × 1) is the vector of the coil-combined k-space of multiple EPI image acquired with a single rEPI readout, which needs to be reconstructed. *N*_1_ and *N*_2_ are the width of k-space along the readout and phase encoding dimensions, *N_e_* is the number of EPI images. *b* (*N*_1_*N*_2_*N*_*c*_/*R* × 1) is the acquired multi-coil k-space data, *N_c_* is the number of receive coils and *R* is the parallel imaging under-sampling factor applied along the phase encoding direction. The operator *A* performs composite operation of *M* ∘ *F* ∘ *S* ∘ *F*^−1^, where *F* and *F*^−1^ are Fourier transform and its inverse, *S* is coil sensitivity encoding and *M* denotes the k space sampling. *H*(*x*) is the block-Hankel structured matrix forming from the multi-echo k-space data (Fig. 2a), which concatenates vectorized k space local patches from multiple EPI images along the column dimension. *r* is the rank parameter of the block-Hankel structure matrix that needs to be pre-defined.

A number of numerical algorithms have been developed to solve structured low-rank constrained MRI reconstruction problems^19,22–25^. Here, a nonconvex formulation for structured low-rank constrained reconstruction was used^19^. The reconstruction described in Eq. 2 is reformulated to the following optimization problem:

Eq.3 $$x=\underset{x}{\text{arg}\;\text{min}}\|Ax-b{\|}_{l_{2}}^{2}+\lambda \text{Φ}(H(x))$$

where Φ(·) is a non-convex regularization that imposes a low rank constraint on the Hankel matrix *H*(*x*):

Eq.4 $$\text{Φ}(H(x))=\underset{Y:rank(Y)\leq r}{\text{min}}\|H(x)-Y{\|}_{F}^{2}$$

where ‖·‖_*F*_ is the Frobenius norm and *λ* is a regularisation parameter balancing the data consistency term and the low rank constraint term. Eq. 3 is solved iteratively to jointly recover k-space data of all EPI images.

### MRI Experiment

The proposed rEPI sequence was implemented on a 3T whole body MRI scanner (Siemens Prisma, Erlangen, Germany). Phantom data and in vivo data from two subjects were acquired to evaluate the performance of the proposed method using a 32-channel receive coil. For comparison, a conventional me-EPI sequence was also implemented and used as a reference. Written informed consent in accordance with local ethics was obtained from each subject.

#### Phantom validation

A spherical water phantom was scanned to evaluate the accuracy of rEPI based ΔB_0_ estimation. The rEPI data was acquired with FOV=220×220×100mm^3^, in-plane resolution=1.7mm×1.7mm, slice thickness=2mm without slice gap, TR=3.6s, flip angle=90°, echo spacing =0.76ms, 3 EPI images were acquired for each readout (Fig. 1c). Parallel imaging under-sampling factors R=1 and 2 were evaluated, with associated TE/TR=54ms/5.85s and 31ms/3.6s, respectively. Conventional single-shot me-EPI data were also acquired with the same FOV and resolution as the rEPI protocol. Two echoes were acquired for me-EPI with TE1/TE2/TR=54ms/151ms/10.7s for R=1 and TE1/TE2/TR=31ms/80.9ms/6.1s for R=2. Posterior-anterior phase encoding was used to avoid signal piling up in the frontal. Coil calibration data were acquired using FLEET method^26^.

#### In vivo data without instructed motion

To assess rEPI based ΔB_0_ estimation and distortion correction on in vivo data, the two subjects were scanned using the same protocols as in the phantom experiment. Subjects were instructed to remain stationary during the acquisition.

#### In vivo data with instructed motion

To investigate the performance of the proposed method in the presence of ΔB_0_ variations, an rEPI time series was acquired from each subject with instructed motion during the scan. 64 volumes were acquired using the same scan parameters as the R=2 rEPI protocol in the phantom experiment. The total scan time was 4min. The subjects were instructed to change their head to different poses and remain still for about 30s after each change. After an initial 20s scan, the head poses used were: rotation with the head-foot axis towards the left, rotation with the head-foot axis towards the right, rotation upwards with the left-right axis, rotation downwards with the left-right axis, rotation with the anterior-posterior axis towards the left shoulder, rotation with the anterior-posterior axis towards the right shoulder. The instruction was given to the subjects in real time via a screen.

For all phantom and in vivo scans, a multi-echo gradient-echo (GRE) based field map was also acquired with FOV=220×220×140mm^3^, in-plane resolution=2.4mm×2.4mm, slice thickness=2mm without slice gap, TE1/TE2/TR=4.86 ms/7.32 ms/682ms. Standard shimming was performed at the beginning of all experiments. For in vivo scans, a T1w structural image was also acquired using an MPRAGE sequence with FOV=256×256×208mm^3^, 1mm isotropic resolution, TE/TR/TI=2.03/2000/880ms and Flip angle=8°.

### Reconstruction implementation

The raw rEPI data were first processed with Nyquist ghost correction and EPI ramp-sampling correction. The reconstruction used the S-matrix style Hankel-structured matrix^19^ and extended it from a multi-coil formulation^27,28^ to multi-coil, multi-echo formulation. Eq. (3) was solved using multiplicative half-quadratic algorithm^29^ for fast computation. To exploit the correlation between coils, the input of the Hankel transform was augmented with a multi-channel formulation by multiplying the reconstructed image with sensitivity maps followed by Fourier transform^27^.

The image reconstruction was performed offline in MATLAB. The kernel size for the Hankel transform was set empirically to 5×5. A fixed number of 15 iterations was used. The multi-channel datasets were compressed from 32 channels to 18 channels for computational efficiency. The regularization parameter *λ* =0.001 and rank number *r* =60 (in vivo data) and 40 (phantom data) were selected based on visual inspection of the reconstruction quality. The reconstructed individual echo images were sum-of-squares combined to produce the final magnitude image. To compare the joint multi-echo reconstruction and conventional parallel imaging methods, all rEPI data were also reconstructed with GRAPPA^18^ and SENSE^17^.

### Field map estimation and distortion correction

For me-EPI data, a phase evolution map ΔΦ(*r*) was calculated from the phase of the two EPI images. For rEPI data with 3 echoes, ΔΦ(*r*) was calculated as

Eq.5 $$\text{ΔΦ}(\text{r})=\frac{{\text{ΔΦ}}_{1,2}(\text{r})+{\text{ΔΦ}}_{2,3}(\text{r})}{2}$$

where ΔΦ_1,2_ and ΔΦ_2,3_ are the phase difference between echo 1 and echo 2, echo 2 and echo 3, respectively. The calculated phase evolution map was phase unwrapped with FSL’s prelude tool and a 4^th^ order polynomial fitting with FSL’s fugue tool^30^. A ΔB(r) map was calculated based on Eq. 1 using the processed ΔΦ(r) and ΔTE. For GRE based field mapping, the field map was calculated using FSL’s fsl_prepare_fieldmap tool^30^.

Distortion correction with me-EPI and rEPI based field maps was performed using SPM’s Fieldmap toolbox (v12)^31^. Distortion correction with GRE field map was performed using fugue ^30^. For in vivo data acquired with instructed motion, the GRE field map was registered to individual EPI image volume via FSL’s Flirt^32,33^ with 6 degrees of freedom, followed by distortion correction.

### Evaluation

For phantom data, the proposed rEPI based method was assessed with respect to the conventional me-EPI. Specifically, the magnitude images of rEPI were evaluated against the first echo image of me-EPI (matched TE with rEPI) using the normalized-root-mean-square-error (nRMSE) metric. The rEPI based ΔB_0_ maps were evaluated against me-EPI based ΔB_0_ maps using the absolute difference.

For in vivo data acquired without instructed motion, rEPI and me-EPI images were registered to the structural image using Flirt (6 degrees of freedom and ‘bbr’ cost) to assess the alignment of distortion-corrected images with respect to the structural image. ΔB_0_ maps calculated from me-EPI, rEPI and GRE sequence were also registered to the structural image. EPI-based ΔB_0_ maps were then evaluated against GRE based ΔB_0_ maps using the absolute difference. The mean and standard deviation values of the absolute difference maps within a brain mask were calculated.

The in vivo rEPI time-series data were aligned to the first volume using Flirt with 6 degrees of freedom. Standard deviation and temporal SNR (tSNR) maps were calculated for different processing approaches: without distortion correction, distortion correction with GRE based static field map and distortion correction with rEPI based dynamic field maps. Image volumes with significant motion corruptions were excluded from the tSNR and standard deviation calculations, including 10 and 12 volumes for subject 1 and 2, respectively.

## Results

**Figure 3:**
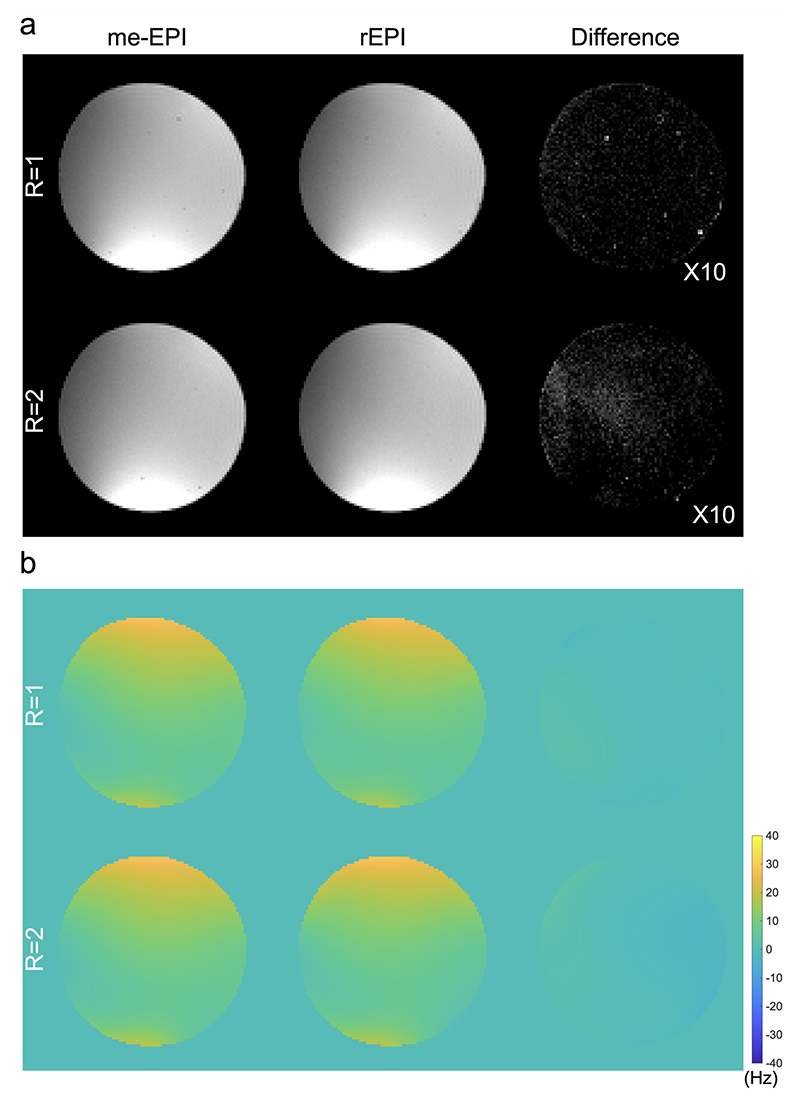
Magnitude images and ΔB_0_ maps acquired with me-EPI and rEPI from the phantom experiment. (a) Magnitude images of rEPI and me-EPI (the 1^st^ echo) are comparable at both under-sampling factors (R=1 and R=2). The difference maps of magnitude images are amplified by 10× for ease of visualization. (b) The ΔB_0_ maps estimated from me-EPI and rEPI data are highly consistent.

Fig. 3 compares the magnitude images and ΔB_0_ maps acquired with me-EPI and rEPI from the phantom experiment. The magnitude images of rEPI and me-EPI (1^st^ echo, matched TE to rEPI) are very similar (Fig. 3a). Using the me-EPI magnitude images as reference, the nRMSE of rEPI magnitude images are 0.015 for R=1 and 0.027 for R=2, respectively. As the 1^st^ echo of me-EPI is equivalent to a conventional single-shot EPI image, this result suggests the proposed rEPI acquisition with associated joint reconstruction can provide a comparable magnitude image as the conventional single-shot EPI. The ΔB_0_ maps estimated from me-EPI and rEPI also demonstrate a good agreement (Fig. 3b) with a mean absolute difference of 0.84Hz for R=1 and 1.81Hz for R=2, corresponding to 0.04 and 0.09 voxel shifts, respectively.

**Figure 4:**
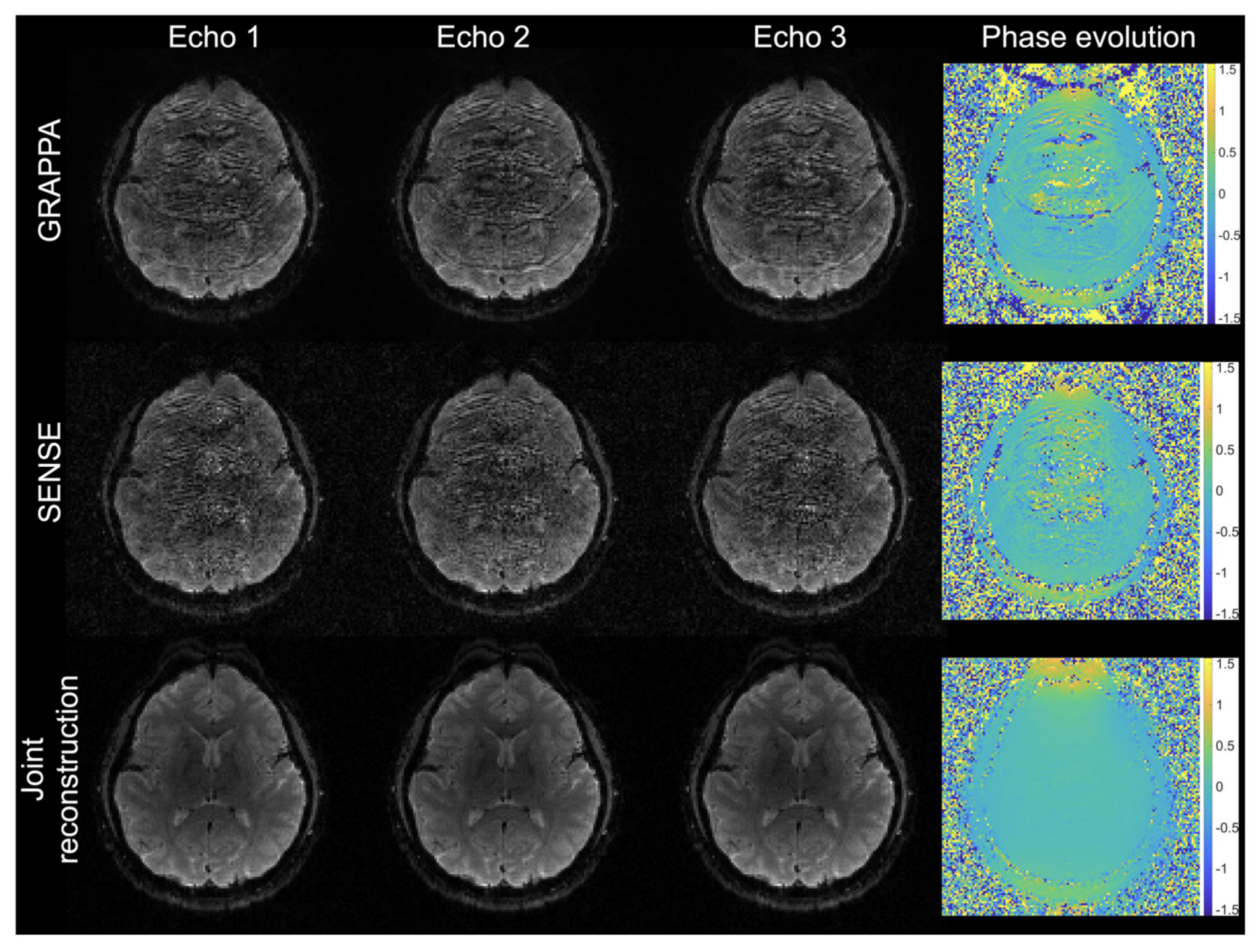
Comparison of conventional parallel image reconstruction (GRAPPA and SENSE) and joint multi-echo reconstruction of rEPI data. Individual echo images reconstructed with GRAPPA and SENSE suffer from strong residual aliasing due to the high under-sampling factor. With joint multi-echo reconstruction, high-quality individual echo images can be reconstructed.

Fig. 4 compares joint multi-echo reconstruction and echo-by-echo parallel imaging reconstruction with GRAPPA and SENSE. rEPI data acquired from one subject with R=2 was used, and the under-sampling factor for each echo is R=6. Individual echo images reconstructed with GRAPPA and SENSE suffer from strong residual aliasing due to the high under-sampling factor. With the joint multi-echo reconstruction, high-quality individual echo images can be reconstructed. The phase evolution map calculated from the GRAPPA and SENSE reconstruction is corrupted by aliasing artifacts and noise, while the joint multi-echo reconstruction provides a clean phase evolution map.

**Figure 5:**
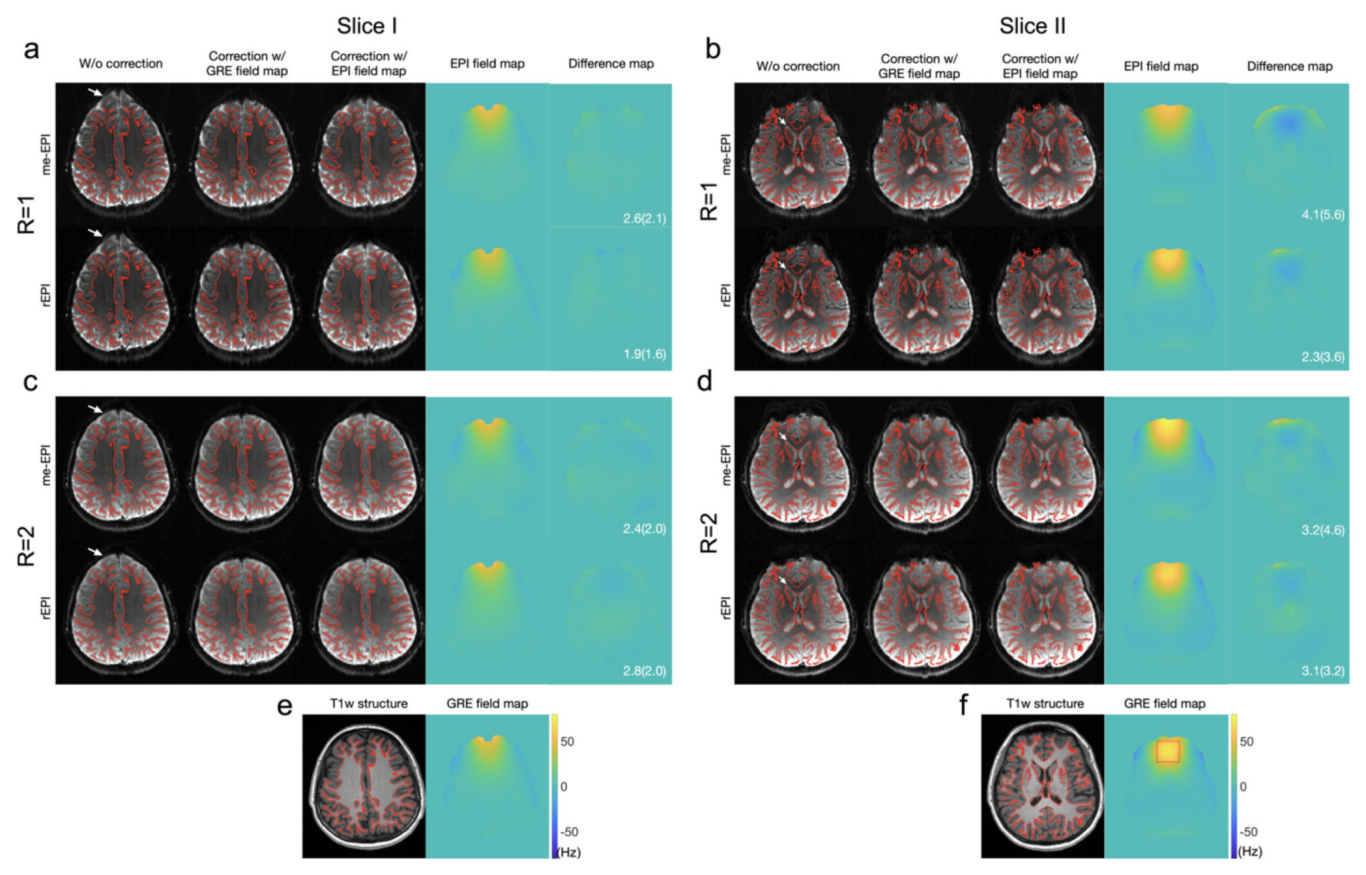
Comparison of ΔB_0_ maps and distortion correction results from a subject scanned without instructed motion. Two brain slices acquired using me-EPI and rEPI with under-sampling factors R=1 and 2 are shown. The magnitude images of me-EPI are from the 1st echo. Consistent with the phantom experiment, the magnitude image of rEPI is very similar to the 1^st^ echo image of me-EPI. With the rEPI based ΔB_0_ map, distortions (indicated by arrows in the uncorrected images) are effectively reduced, comparable to GRE field map based correction. Difference between EPI-based ΔB_0_ maps and GRE based ΔB_0_ maps are shown. The mean and standard deviation values of the absolute difference maps within a brain mask are also listed. White matter boundaries (red edges) were extracted from the structural image and overlaid on each EPI image for visual evaluation of distortion correction performance. A ROI in the frontal area (f, GRE field map) in slice II indicates a region where ΔB_0_ derived from me-EPI with R=1 has a large bias.

Fig. 5 compares the ΔB_0_ maps and distortion correction results from a subject scanned without instructed motion. Consistent with the phantom experiment, the magnitude images of rEPI and me-EPI (1^st^ echo) are very similar. With rEPI based ΔB_0_ map, distortions (indicated by arrows in the uncorrected images) are effectively reduced, with comparable quality to GRE field map based corrections. Compared to me-EPI, rEPI achieves a much shorter TR (3.6s versus 6.1s with R=2) for the same spatial resolution, which enables a higher temporal resolution for fMRI acquisition. The ΔB_0_ maps estimated with rEPI are generally in good agreement with GRE field maps with consistent spatial patterns and off-resonance frequency ranges. Note that the ΔB_0_ maps estimated with EPI-based methods and GRE-based method are different as they are in the distorted and undistorted spaces, respectively.

In Fig. 5, me-EPI based ΔB_0_ map estimation generally provides a comparable accuracy as rEPI with similar mean difference. However, for R=1 at Slice II (Fig. 5b), me-EPI based ΔB_0_ map has a higher error level. Particularly, in the frontal area, the mean (standard deviation) of ΔB_0_ within a ROI located in the frontal region of slice II (Fig. 5f, red rectangle) are 44.85 (7.06) for me-EPI with R=1, 59.48 (9.73) for me-EPI with R=2, 54.07 (8.71) for rEPI with R=1, 55.37 (7.51) for rEPI with R=2 and 58.90 (8.35) for GRE field map. The stronger bias of ΔB_0_ estimation from me-EPI with R=1 is likely due to the low SNR of later echoes and phase unwrapping errors in the phase evolution map ΔΦ(*r*). The magnitude and phase images of the 2^nd^ echo of me-EPI with R=1 at Slice II are shown in Supporting Information Figure S1, demonstrating significant phase wrapping and noisy phase estimation. The bias of ΔB_0_ estimation with me-EPI also presents in the data acquired from the other subject (Supporting Information Figure S2).

**Figure 6:**
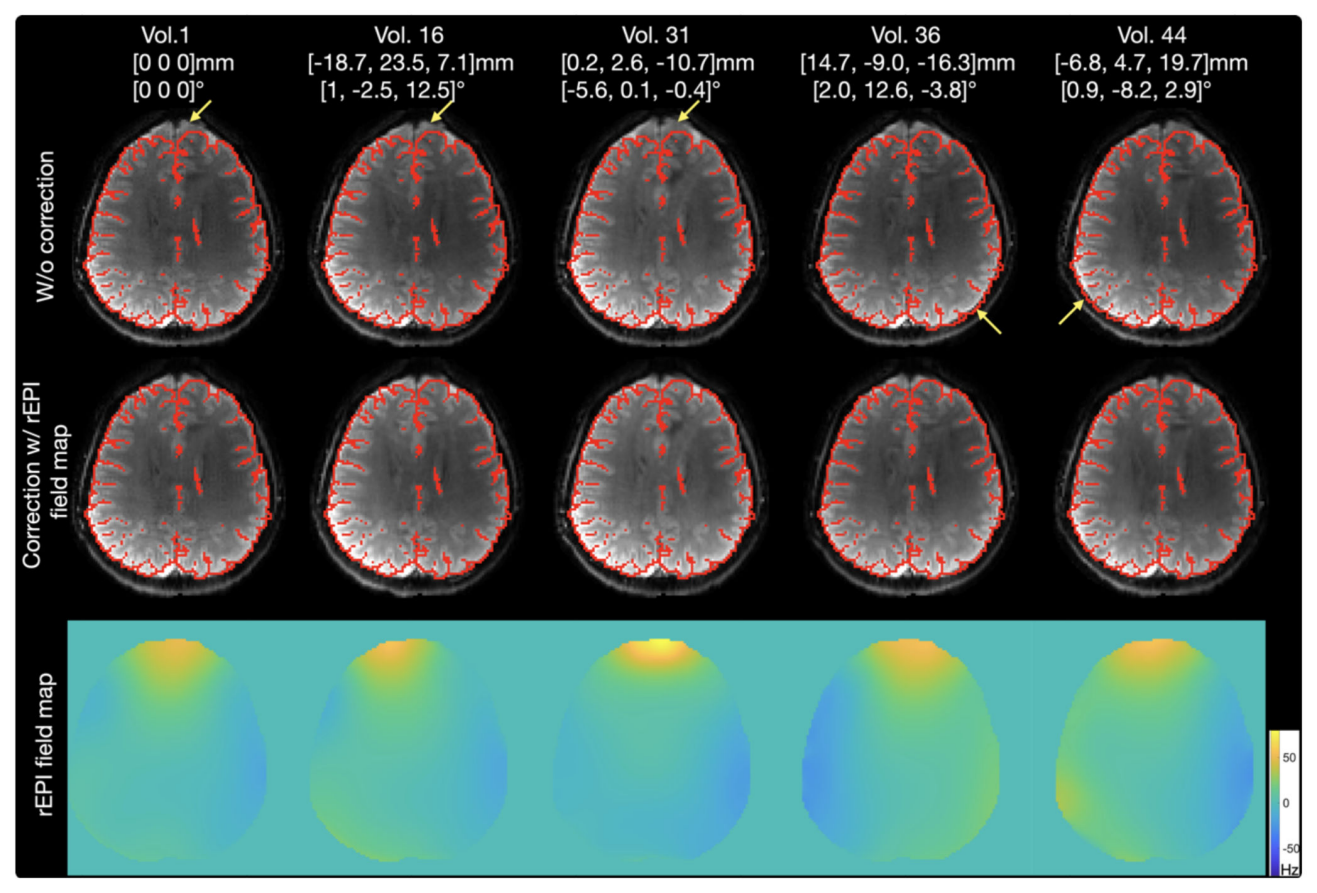
Dynamic rEPI images and estimated ΔB_0_ maps from a subject scanned with instructed motion. Five volumes acquired with different head poses are shown. Motion parameters for each volume are also listed. All volumes are registered to the first volume using FSL’s flirt with 6 degrees of freedom. Red outlines are gray matter and CSF boundaries generated from the structural data and registered to the first rEPI volume. Due to subject motion, ΔB_0_ changes across volumes, leading to temporally varying image distortions (indicated by the yellow arrows). With rEPI based ΔB_0_ estimation, the dynamic distortions in the image time series are robustly corrected.

**Figure 7:**
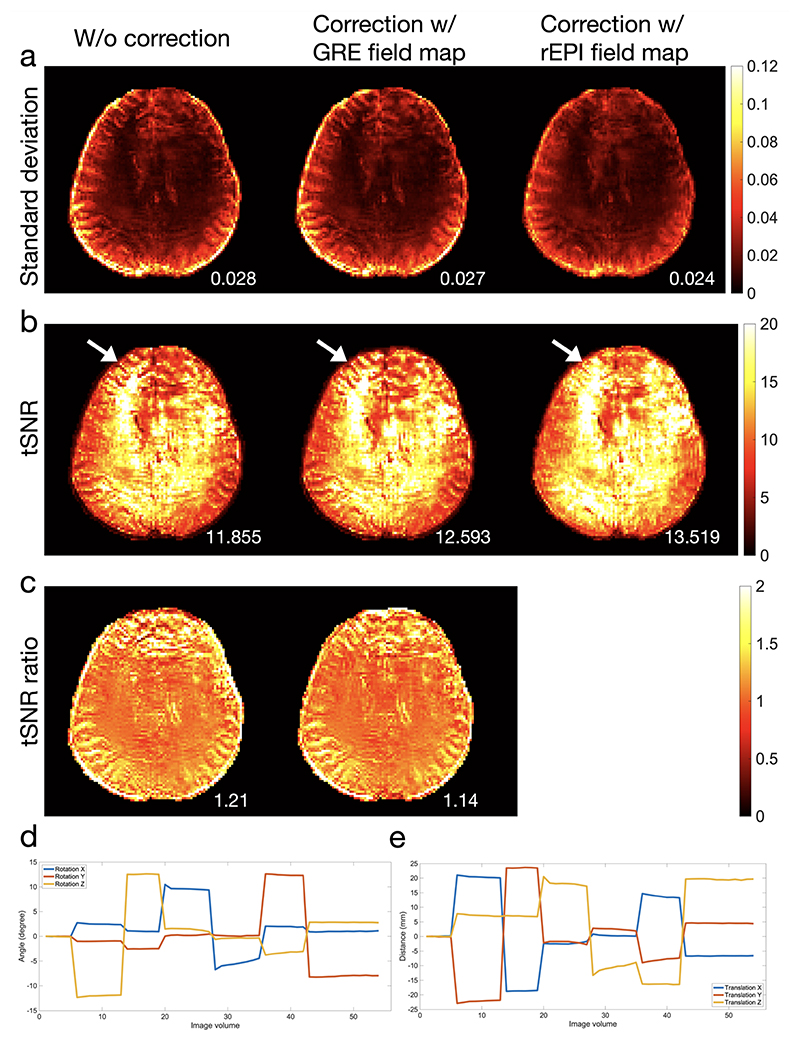
Standard deviation and tSNR of the rEPI time series from a subject scanned with instructed motion. Maps of standard deviation (a) and tSNR (b) of uncorrected time series, correction with GRE-based static field mapping and correction with rEPI-based dynamic field mapping are shown. (c) The relative tSNR (‘tSNR ratio’) of the time-series corrected with rEPI-based dynamic field mapping compared to the tSNR of uncorrected time-series and the tSNR of time-series corrected with GRE-based static field mapping. Rotation (d) and translation (e) motion estimated from the data are also shown. The averaged standard deviation, tSNR and tSNR ratio across the brain are calculated and shown in each image. Distortion correction with rEPI based dynamic field mapping achieves higher tSNR and lower standard deviation than the correction with GRE based static field mapping.

Fig. 6 shows rEPI based dynamic ΔB_0_ estimation and distortion correction from a subject scanned with instructed motion. Five volumes acquired with different head poses are shown. Due to subject motion, ΔB_0_ changes across volumes, leading to temporally varying image distortions (indicated by the yellow arrows). rEPI based ΔB_0_ estimation can capture dynamic ΔB_0_ variations and provide robust correction for all volumes. Fig. 7 shows the time series analysis of the same data. The time series without distortion correction demonstrates the highest standard deviation and the lowest tSNR. Distortion correction with GRE based static field mapping provides improvement over the uncorrected data, but it cannot provide accurate correction due to motion induced ΔB_0_ field variations. rEPI based dynamic field mapping estimates a ΔB_0_ map for each image volume, which captures the dynamic ΔB_0_ variations and enables more robust correction of image distortions. As demonstrated in the standard deviation and tSNR maps, the correction with rEPI based dynamic field mapping achieves lower standard deviation and higher tSNR compared to the correction with GRE based static field mapping (averaged tSNR ratio=1.14) and the uncorrected data (averaged tSNR ratio=1.21), particularly at tissue boundaries (indicated by arrows). Data from the other subject (Supporting Information Figure S3) shows consistent results.

## Discussion and conclusions

This work develops a new method for dynamic ΔB_0_ mapping and distortion correction using single-shot rEPI acquisition and joint multi-echo reconstruction. The proposed method can generate a 2D ΔB_0_ map from a single readout, providing high robustness against subject motion and respiration, which are the primary sources of ΔB_0_ field fluctuations during fMRI scans. Compared to conventional me-EPI, the proposed rEPI trajectory acquires multiple EPI images with a very short ΔTE, which allows accurate ΔB_0_ estimation without limiting the spatial and temporal resolution. In vivo experiments with instructed motion show that rEPI based dynamic field mapping and distortion correction can provide improved temporal stability of EPI time series than GRE based static field mapping.

For multi-echo sequence based ΔB_0_ mapping, using a too large ΔTE can lead to signal loss in later echoes and phase unwrapping errors, while using a too small ΔTE can lead to low phase evolution that is prone to noise corruption. The optimum ΔTE for ΔB_0_ mapping depends on the SNR of the data and ΔB_0_ inhomogeneity, which can be highly variable across image protocols, field strengths and subjects. In this work, the 3-echo rEPI sequence is used with a ΔTE of 1.52ms, which is close to what is routinely used (2.45ms) in our center for ΔB_0_ mapping with multi-echo gradient echo sequence. This choice is expected to provide a reliable ΔB_0_ estimation for different scan conditions. In addition, with this ΔTE, the multiple EPI echoes acquired with rEPI retain a highly similar image contrast which is ideal for effective joint multi-echo reconstruction.

**Figure 8:**
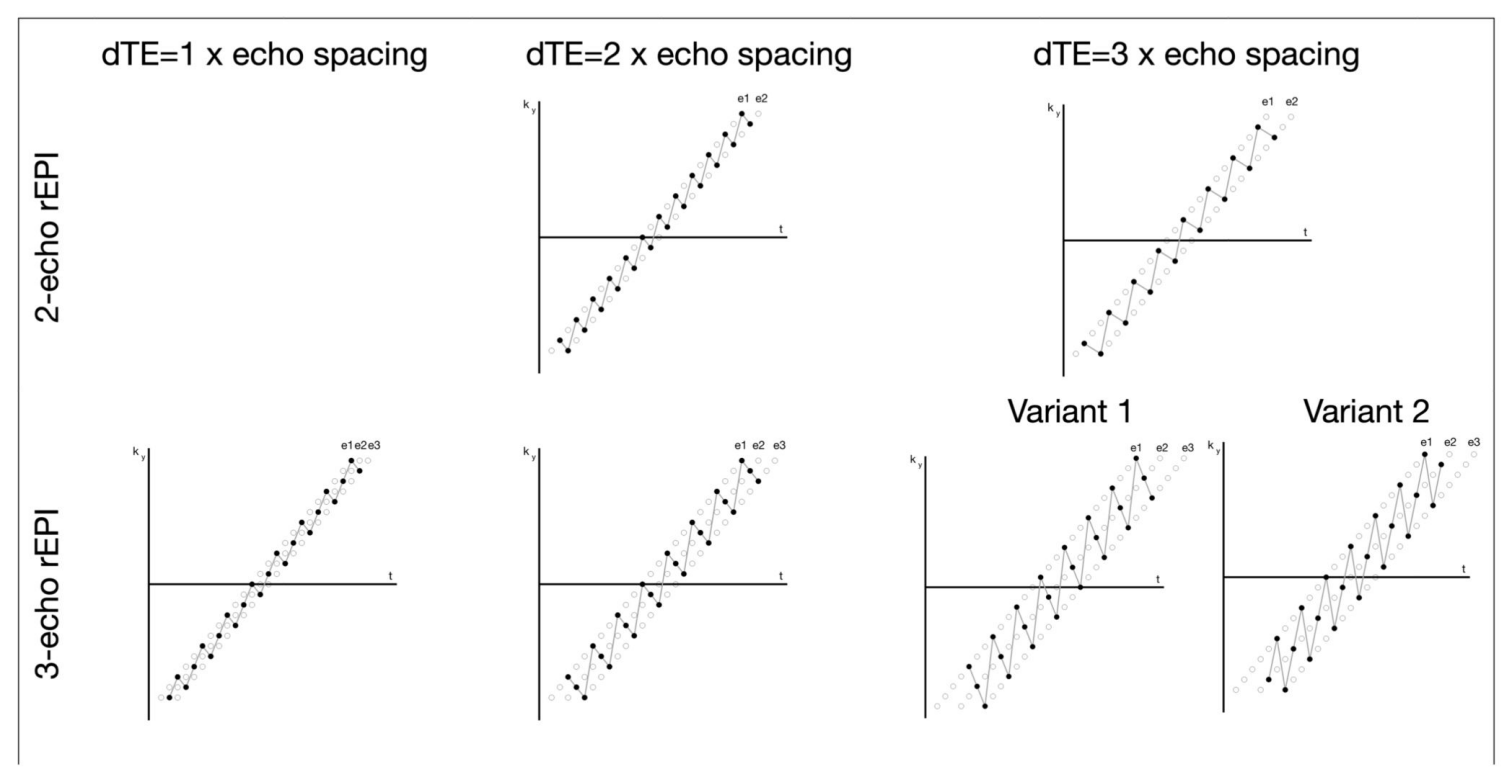
rEPI trajectories designed with 2- and 3-echoes, with ΔTE=1/2/3 × echo spacing, demonstrated in k_y_-t space. For 2-echo rEPI with ΔTE =1 × echo spacing, no feasible trajectory exists based on the two design principles. For 3-echo rEPI with ΔTE = 3 × echo spacing, more than one possible trajectory exists.

Besides the rEPI trajectory shown in Figure 1, alternative rEPI trajectories with different numbers of echoes and different ΔTEs can also be designed. To improve distortion correction and joint multi-echo reconstruction, two design principles can be considered in the rEPI trajectory design. Firstly, the multiple EPI images acquired with an rEPI readout use the same under-sampling factor to ensure consistent image distortion across multiple echoes. Secondly, the multiple EPI images acquired with an rEPI readout sample complementary k_y_ lines (e.g., every sampled point in the k_y_ -t space is only sampled once by an rEPI readout), to make better use of the coil sensitivity in the joint multi-echo reconstruction. Exemplar rEPI trajectories with 2 and 3 echoes, and different echo spacing are shown in Fig. 8. It is worth noting that, for some configurations, there might be no available trajectory based on the two principles listed above (e.g., 2-echo rEPI with ΔTE =1 × echo spacing). For some configurations, there may exist more than one trajectory (e.g., 3-echo rEPI with ΔTE = 3 × echo spacing).

The 2-echo rEPI trajectories were considered initially, as two echoes are sufficient for ΔB_0_ estimation. However, acquiring an even number of echoes with rEPI (e.g., 2-echo rEPI) are prone to residual phase errors between odd and even EPI readout lines, as each image is acquired with the same readout gradient polarity. Recent work based on readout-reversed first echo^13^ demonstrated the capability to correct for EPI odd-even phase errors for fMRI time series, which can be potentially integrated with rEPI to enable robust ΔB_0_ estimation with an even number of echoes. Note that conventional me-EPI with under-sampling can be considered as a special case of rEPI with a large rewinder blip that traverses from -k_y_max_ to k_y_max_ in a single step.

It is worth noting that, the previously developed IDEA-EPI method also acquires two echoes with a single EPI readout and ΔTE = 1 × echo spacing, which have been applied to EPI Nyquist ghost correction^34^ and dynamic distortion correction^35^. The main difference between IDEA-EPI and 2-echo rEPI is that IDEA-EPI acquires the same ky lines for both echoes, while 2-echo rEPI acquires complementary ky lines across echoes, which allows more effective joint multi-echo reconstruction.

Compared with conventional single-shot EPI, the minimum achievable echo spacing and TE of rEPI may be increased due to the use of rewinder blips. This effect was investigated by comparing rEPI and conventional single-shot EPI with the imaging protocols used in this work. Our scanner can operate at different gradient modes with different maximum gradient amplitudes and slew rates. Using the ‘fast’ mode, the minimum achievable echo spacings with rEPI are 0.73ms (R=1) and 0.76ms (R=2), and with conventional single-shot EPI are 0.72ms for both R=1 and R=2. The minimum achievable TEs with rEPI are 52ms (R=1) and 30ms(R=2), and with conventional single-shot EPI are 52ms (R=1) and 29ms (R=2). Using the more powerful ‘performance’ mode, the minimum achievable echo spacings with rEPI are 0.63ms (R=1) and 0.66ms (R=2), and with conventional single-shot EPI are 0.6ms for both R=1 and R=2. The minimum achievable TEs with rEPI are 45ms (R=1) and 26ms (R=2), and with conventional single-shot EPI are 43ms (R=1) and 24ms (R=2). The small difference between rEPI and conventional single-shot EPI in the achievable minimum echo spacing and TE indicates the impact of blip rewinder on sequence timing is not significant for the protocols studied here. Another effect associated with the rewinder blips is increased eddy currents, which may introduce artifacts in the image. In the data acquired in this work, no additional eddy currents-related artifacts were observed in rEPI data compared to conventional single-shot EPI. However, using very large rewinder blips can lead to a strong eddy current effect, which may introduce artifacts in the image.

The joint multi-echo reconstruction provides significantly improved reconstruction than conventional parallel imaging methods (Fig. 4) by exploiting shared information between EPI images from a single rEPI readout. As the final magnitude image of rEPI combines all individual EPI images, its SNR is expected to be comparable to that of a conventional single-shot EPI image acquired with matched scan parameters, which is consistent with the phantom and in vivo results in this work. The joint multi-echo reconstruction method can also be applied to other multi-echo reconstruction problems as well, including me-EPI. However, compared to me-EPI, rEPI allows more effective joint multi-echo reconstruction as the multiple echo images with rEPI are more similar to each other thanks to the much shorter ΔTE. A comparison of joint multi-echo reconstruction of me-EPI and rEPI with matched under-sampling factor and sampling patterns is shown in Supporting Information Figure S4. The joint multi-echo reconstruction of me-EPI data outperforms the conventional parallel imaging reconstruction (Fig.4), but still suffers from significant residual artIfacts. In comparison, joint multi-echo reconstruction of rEPI data provides much improved image quality.

The reconstruction of in vivo time series data assumes coil sensitivities are sufficiently smooth such that brain motion relative to the stationary coils would not cause a significant impact in the reconstruction. However, this assumption may not hold with large motions. To address this challenge, methods including prospective motion correction^36,37^ and motion-compensated reconstruction^38^ can be used for robust reconstruction. Nevertheless, the main finding in these experiments is still valid, which shows rEPI based dynamic field mapping can provide improved temporal stability than GRE based static field mapping method.

Recently, the Echo Planar Time-resolved Imaging (EPTI) method has been proposed, which is a multi-echo acquisition technique that can provide time-resolved distortion-free images at different TEs throughout the EPI readout window^39^. To correct for inter-shot phase difference due to dynamic ΔB_0_ variations, a greedy search-based algorithm is used in EPTI to estimate shot-to-shot ΔB_0_ variations, together with a low order polynomial modelling of the ΔB_0_ variation maps. The proposed rEPI method directly measures dynamic ΔB_0_ maps from multiple EPI images from a single readout and imposes no model constraints on ΔB_0_ in image reconstruction, which allows it to capture high order field fluctuations that can present at high field strengths and with subject motion. Integration of the concept of rEPI sampling with EPTI may further improve the robustness of EPTI method to dynamic ΔB_0_ changes, which is an interesting research direction to explore in the future.

Besides dynamic ΔB_0_ mapping, multi-echo EPI can also be applied for BOLD contrast optimization^40^ and differentiation of BOLD and non-BOLD signal^41^, which have been demonstrated using conventional me-EPI. Although the proposed rEPI method can provide multiple echoes, the short echo time difference required by the joint multi-echo reconstruction limits the signal magnitude contrast between echoes. Therefore, with its current implementation, rEPI is not considered as an ideal option for those applications.

Here, unwrapping of me-EPI data was performed with Prelude. Recently, more advanced unwrapping tools have been developed^42^, which may provide more accurate unwrapping for me-EPI data. However, given the long echo time difference, the unwrapping error would always be a challenge for me-EPI, particularly for high resolution scan at high field strengths.

This work presents a proof of principle of rEPI based dynamic ΔB_0_ mapping and distortion correction with a gradient echo sequence at 3T, although it is straightforward to extend rEPI to other sequences utilizing an EPI readout (e.g., diffusion imaging, perfusion imaging and spin-echo fMRI) and field strengths. In addition, the rEPI trajectory is compatible with simultaneous multi-slice acquisition with Blipped-CAIPI sampling^43^ as the modifications of gradient blips are applied on different axes for these two approaches. This integration could enable dynamic ΔB_0_ mapping with a higher scan efficiency.

## Supplementary Material

Supplementary Material

## Data Availability

The reconstruction code is available online at https://github.com/WWimaging/JointMErecon
