# Supplementary material for: Dynamic field mapping and distortion correction using single-shot blip-rewound EPI (rEPI) and joint multi-echo reconstruction
Source: Magn Reson Med. Author manuscript; Available in PMC 2025 Oct 6. (PMC7618198; doi:10.1002/mrm.30038)
Supplement: Supplementary Material [file EMS209191-supplement-Supplementary_Material.pdf]

Supporting Information

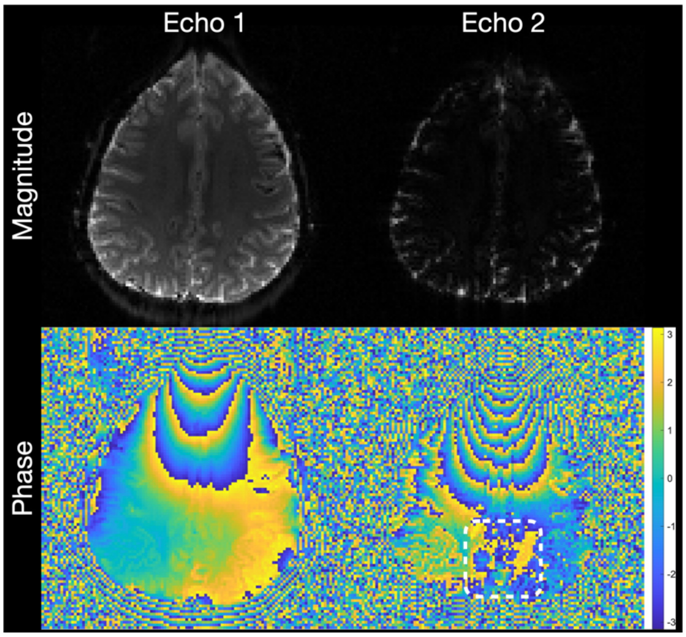

Supporting Information Figure S1. Magnitude and phase images acquired from one subject using me-EPI with R=1. The TE of the 2nd echo is very long (151ms), which leads to strong phase wrapping and noisy phase estimation (e.g., noise corruption in the region indicated by the dashed rectangle) that can bias the ΔB_0 estimation.

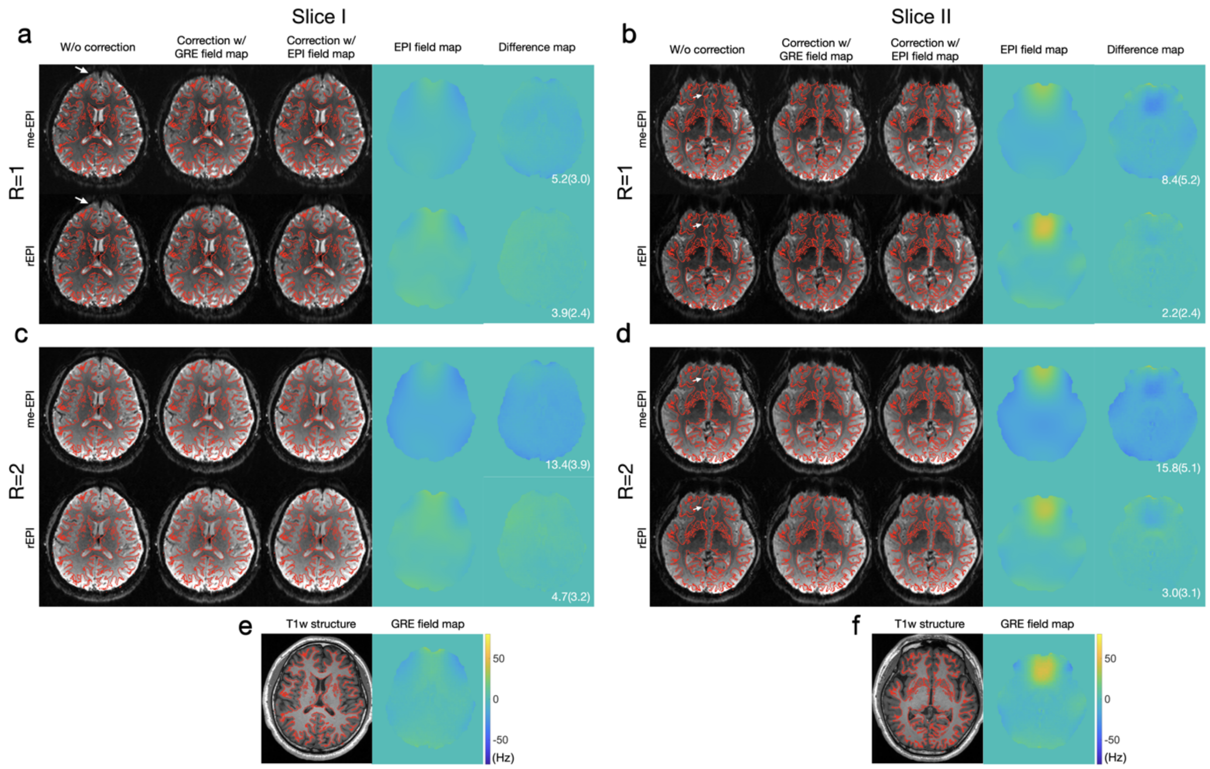

Supporting Information Figure S2. Comparison of ΔB_0 maps and distortion correction results from the other subject scanned without instructed motion. Two brain slices acquired using me-EPI and rEPI with under-sampling factors R=1 and 2 are shown. The magnitude images of me-EPI are from the 1st echo. Consistent with the phantom experiment, the magnitude image of rEPI is very similar to the 1st echo image of me-EPI. With the rEPI based ΔB_0 map, distortions (indicated by arrows in the uncorrected images) are effectively reduced, comparable to GRE field map based correction. Difference between EPI-based ΔB_0 maps and GRE based ΔB_0 maps are shown. The mean and standard deviation values of the absolute difference maps within a brain mask are also listed. In this subject, ΔB_0 maps derived from me-EPI show a negative bias compared to rEPI and GRE based ΔB_0 maps. White matter boundaries (red edges) were extracted from the structural image and overlaid on each EPI image for visual evaluation of distortion correction performance.

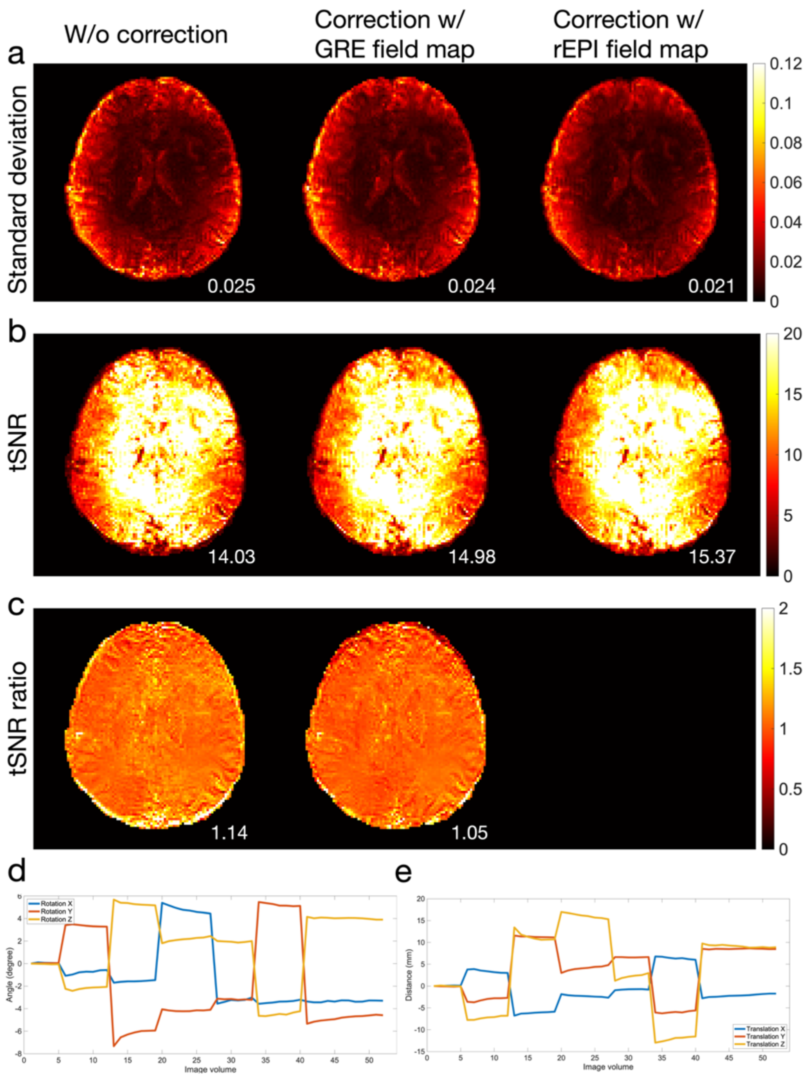

Supporting Information Figure S3. Standard deviation and tSNR of the rEPI time series from the other subject scanned with instructed motion. Maps of standard deviation (a) and tSNR (b) of uncorrected time series, correction with GRE-based static field mapping and correction with rEPI-based dynamic field mapping are shown. (c) The relative tSNR ('tSNR ratio') of the time-series corrected with rEPI-based dynamic field mapping compared to the tSNR of uncorrected time-series and the tSNR of time-series corrected with GRE-based static field mapping. Rotation (d) and translation (e) motion estimated from the data are also shown. The averaged standard deviation, tSNR and tSNR ratio across the brain are calculated and shown in each image. Distortion correction with rEPI based dynamic field mapping achieves higher tSNR and lower standard deviation than the correction with GRE based static field mapping.

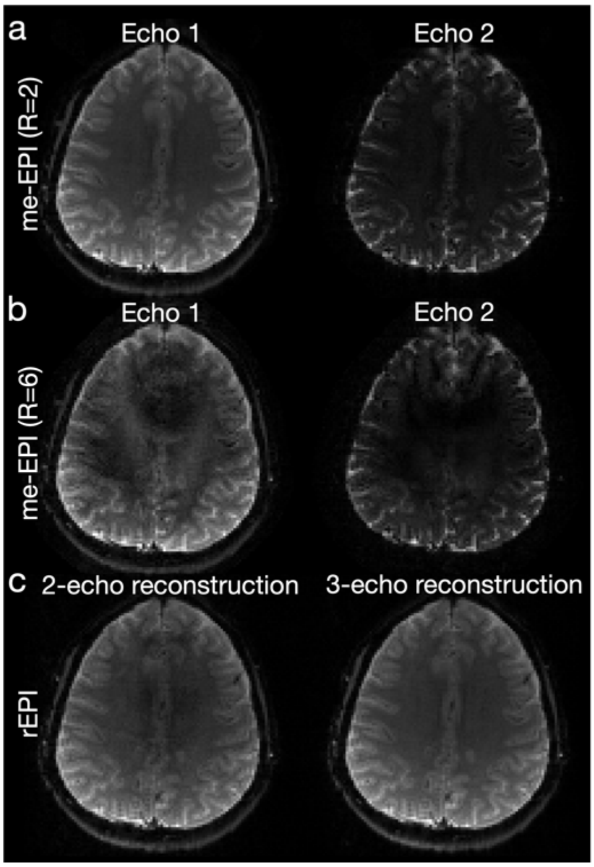

Supporting Information Figure S4. Comparison of joint multi-echo reconstruction of me-EPI and rEPI data. (a) me-EPI images with R=2, which are used as a reference. The echo time difference is 49.9ms (b) Joint 2 echo reconstruction of me-EPI data with R=6. The me-EPI were generated by retrospectively under-sampling the me-EPI data in (a) by a factor of 3. The sampling patterns of the two echoes are complementary and the same as the first two echoes of rEPI in (c). The echo time difference is the same as (a). Although the joint multi-echo reconstruction of me-EPI data is better than those with conventional parallel imaging methods (Fig. 4), there are still significant residual artifacts. (c) Joint multi-echo reconstruction of rEPI data with R=2. The under-sampling factor of each echo is R=6, the same as me-EPI in (b). The echo time difference is 1.52 ms for rEPI. Joint reconstruction of the first two echoes ('2-echo reconstruction') outperforms that of me-EPI despite matched sampling patterns and under-sampling factors. This is due to the much shorter echo time difference of rEPI that allows more effective multi-echo reconstruction. By jointly reconstructing all three echoes ('3-echo reconstruction') of rEPI data, the image quality is highly comparable to the reference (echo 1 in (a)).
